# Active Cervical Range of Motion in Babies with Positional Plagiocephaly: Analytical Cross-Sectional Study

**DOI:** 10.3390/children8121146

**Published:** 2021-12-06

**Authors:** Iñaki Pastor-Pons, María Orosia Lucha-López, Marta Barrau-Lalmolda, Iñaki Rodes-Pastor, Ángel Luis Rodríguez-Fernández, César Hidalgo-García, José Miguel Tricás-Moreno

**Affiliations:** 1 Instituto de Terapias Integrativas, 50001 Zaragoza, Spain; inakipas@gmail.com (I.P.-P.); martabarraulalmolda@gmail.com (M.B.-L.); ikirodes95@gmail.com (I.R.-P.); 2 Departamento de Fisiatría y Enfermería, Unidad de Investigación en Fisioterapia, Facultad de Ciencias de la Salud, Universidad de Zaragoza, 50009 Zaragoza, Spain; hidalgo@unizar.es (C.H.-G.); jmtricas@unizar.es (J.M.T.-M.); 3 Departamento de Fisioterapia, Facultad de Medicina, Universidad San Pablo CEU, 28925 Alcorcón, Spain; alrodfer@ceu.es

**Keywords:** positional plagiocephaly, cervical range of motion, supine position

## Abstract

Positional plagiocephaly (PP) is a general term describing cranial distortion from pre- or postnatal forces on the infant head. Abnormal intrauterine forces, multiple births, primiparous mothers, obstetric interventions, prematurity, male sex, excessive time lying in the supine position, and mobility restrictions of the cervical spine have been considered as the main predisposing factors. The objective was to investigate the association between the severity of PP and the active cervical rotation and to analyze the influence of predisposing factors in babies with PP. An analytical cross-sectional study was performed on 74 babies with moderate PP. Clinical and demographic data, cranial vault asymmetry, and active cervical rotation range of motion (ROM) were measured. Associations were analyzed with generalized linear models. The mean age was 16.8 ± 5.0 weeks, and 56.8% were male. A restriction in the ROM of active cervical rotation, especially to the left side, was observed. Our models showed that cranial asymmetry was related with left active cervical rotation ROM (*p* = 0.034) and with being transported in a pushchair (*p* < 0.001). Conclusions: An increased severity of PP was related with being transported in a baby pushchair and with a reduced active cervical rotation ROM toward the most restricted side.

## 1. Introduction

Positional plagiocephaly (PP) is a pathology describing cranial distortion from pre- or postnatal forces on the infant head. PP features are asymmetrical occipital flattening accompanied by anterior displacement of the ear on the same side, often ipsilateral frontal protuberance, and contralateral parietal protuberance with frontal flattening [1]. Prevalence data are reported with a wide range. The data reported in the literature vary from low (13–16%) [2,3,4] and medium, (20–30%) [5,6] to very high (61%) [7] percentages of prevalence in babies. The great variability in prevalence is due to the lack of homogeneity in the diagnostic criteria and the wide age range in which it is studied. Some authors have stated that below the age of three months, PP could be found in almost half of infants [8]. Others studied the prevalence of plagiocephaly and/or brachycephaly between newborns and infants of two years, and they observed 16.0%, 19.7%, 9.2%, 6.8%, and 3.3% prevalence at 6 weeks and 4, 8, 12, and 24 months, respectively [4]. The peak can be observed at four months, and percentages are low after 8 months. Many cases of PP improve over time, but scientific evidence suggests that conservative management strategies can safely and effectively minimize the degree of cranial asymmetry [9,10,11,12].

PP, also known as deformational plagiocephaly, is not caused by the premature closing of a suture, and it is related with non-synostotic factors [13]. Many intrinsic and extrinsic predisposing factors can play a role before, during, and after childbirth. Limited intrauterine space or forced abnormal intrauterine positioning, multiple births, a primiparous mother, obstetric interventions, high birth weight, prematurity, male sex, excessive time lying in the supine position, and mobility restrictions of the cervical spine have been discussed as some of the main factors [14]. However, the etiology of plagiocephaly is not completely clarified, and it is advisable to increase the available evidence on predisposing factors [15].

After the recommendation by the American Academy of Pediatrics (AAP, 1992) to let children lie in the supine position, a noticeable reduction (up to a 50%) in sudden death syndrome was found [16]. However, an increase in non-synostosic PP was also observed [17,18]. Recommendations to maintain supervised wakeful prone “tummy time” during infancy have been established to prevent positional plagiocephaly [19]. Wakeful tummy time has also shown developmental benefits for gross motor skills [20].

Rogers et al. (2009) showed that there was a restriction of active cervical rotation in children in almost all of the plagiocephaly cases, even without a previous diagnosis of congenital muscular torticollis [21]. In fact, the right–left ratio in the posterior flattening is 2:1 in PP, probably due to the preferential position of head contact on the right side, which is associated with a higher range of motion in right cervical rotation and a restriction in left rotation in most newborns [22].

The objective of the present study was to investigate the association between the severity of PP and the active cervical rotation and to analyze the potential influence of prenatal, perinatal, and postnatal predisposing factors described in the literature in a sample of babies with PP.

## 2. Materials and Methods

A non-experimental analytical cross-sectional study with ex post facto multivariate analysis was performed.

The Ethics Committee at the Health Sciences Institute of Aragon approved the study (Registry No. C.P-C.I. PI16/0275; Date: 25 October 2017).

### 2.1. Subjects

A cohort of 74 subjects were recruited after being referred by pediatricians from Sector III of the Public Health Service of Aragon.

The inclusion criteria were: babies under 32 weeks old [4] and infants with at least moderate PP (at least 5 mm of difference between cranial diagonal diameters [23]). Subjects with craniosynostosis and with genetic, infectious, metabolic, or neurological diseases were excluded.

For the calculation of the sample size, we used the cranial vault asymmetry index (CVAI) as the primary outcome measure and descriptive data of the CVAI (9.14% ± 2.98) from a previous study on subjects with similar characteristics [24]. The sample size was calculated using the GRANMO calculator (https://www.imim.cat/ofertadeserveis/software-public/granmo/, accessed on 1 October 2021) [25] with the selection of means, the population estimation option, a confidence level of 0.95, a reference population size that assumed an infinite population, an estimated standard deviation of 2.98, and a desired precision of 0.7. A minimal number of 70 subjects was obtained. The estimated proportion of replacements was 0%.

An informative document about the study was provided to the parents, and an informed consent form was signed after they had read the document and their questions about the study had been answered. Regulations and guidelines regarding understandable and complete information, confidentiality, disclosure of economic interests, absence of coercion, freedom, and acceptance were followed [26].

### 2.2. Measurements

#### 2.2.1. Clinical and Demographic Data

Clinical and demographic data were extracted from the medical history and the testimony of the parents: sex, age (weeks), birth weight (g), firstborn, multiple birth, prematurity (defined as being born during or before the 37th week), instrumental delivery, plagiocephaly side, transport type, and time that the infant spent awake in the prone position and watched at 1 month (min).

#### 2.2.2. Anthropometric Measurements

The following anthropometric parameters were measured: diagonal cranial diameters taken from the frontozygomatic suture (fz) to the contralateral lambdoid suture (lb) [27]. These cranial anthropometric measurements have shown an excellent interrater and intrarater reliability [28]. From these data, the next variable was calculated.

The CVAI was calculated using the formula “[Long diagonal cranial diameter (mm) –Short diagonal cranial diameter (mm)]/Short diagonal cranial diameter × 100” [29]. The classification of the plagiocephaly severity scale (Children’s Healthcare of Atlanta, 2015) [30] is based on the CVAI, and it describes the following levels: level 1: <3.5%; level 2: 3.5 to 6.25%; level 3: 6.25 to 8.75%; level 4: 8.75 to 11.0%; level 5: >11.0%. The CVAI was established as the primary outcome measure.

#### 2.2.3. Active Cervical Rotation ROM Measurement

The active cervical rotation ROM was measured in each direction and was calculated considering the center of the neck as the axis of rotation. The final angle of movement was measured with respect to a line marked on the chair used by the baby, and the line in the crane was marked by a felt strip that joined the most anterior part at the height of the nose and the most posterior part, fixed in a cloth crown. Although Murgia et al. used supine lying for this measurement [31], an upright position was chosen for the measurement in order to be able to observe the control of the head in the sagittal plane.

The baby was sat in a low chair with their upper trunk held under the shoulders by one of their parents. An examiner stood in front of the child and stimulated them by moving a sonorous toy in a semicircle around the child to provoke the rotation of the head until they reached the limit in each direction. Several repetitions on each side were performed to ensure the maximal rotation. The active cervical rotation ROM to each side was registered by a photographic image from above [32].

The photographic image was analyzed with the program GeoCebra v.6 in order to measure active cervical rotation ROM in degrees (°). This measurement has shown a good reliability [33]. Clinical and demographic data and the anthropometric and active cervical rotation (ROM) measurements were registered in the same evaluation session.

### 2.3. Statistical Analyses

The numerical analysis was performed using SPSS 25.0 for Mac.

A descriptive analysis of qualitative variables, offering the absolute frequencies and the percentages in each category, and of quantitative variables, offering the mean ± standard deviation, was carried out.

To model the CVAI as a function of active cervical rotation ROM and the risk factors, three generalized linear models (GLMs) were used. The model type used was that of the main effects with Gamma distribution with the log link function. The parameter estimation method was the hybrid method with the robust estimator.

The first model included total active cervical rotation ROM as a covariate, the second model included the right active cervical rotation ROM as a covariate, and the third model included the left active cervical rotation ROM as a covariate. The fixed continuous covariates for the three models were age, birth weight, and time in prone position at 1 month. The fixed factors (categorical variables with two levels) for the three models were sex (male/female), firstborn (yes/no), multiple birth (yes/no), prematurity (yes/no), instrumental delivery (yes/no), and transport type (pushchair/baby backpack).

Model assumptions were verified. Homoscedasticity was verified by plotting deviance residuals versus fitted values, normality of deviance residuals by a normal probability plot, and collinearity diagnostics with the Variance Inflation Factor (VIF) and the Tolerance Statistic.

Statistical significance was set at *p* < 0.05.

## 3. Results

A total of 74 subjects were incorporated in the study. The mean age of the subjects at the time of measurement was 16.8 ± 5.0 weeks (Table 1); 43.2% were female and 56.8% were male (Table 1). Descriptive values of demographic characteristics, active cervical rotation ROM, and anthropometric measurements can be consulted in Table 1.

## 4. Discussion

The sample of this study consisted of 74 children with PP, with a difference of at least 5 mm between diagonal cranial diameters, that is, children with at least moderate deformity [23]. The GLM outcomes showed that the severity of PP was related to the left active cervical rotation ROM and with transport type independently of the active cervical rotation ROM.

The mean age of the subjects at the time of measurement was 16.8 ± 5.0 weeks (prevalence of positional skull deformity generally peaks at 4 months [34]); 43.2% were female and 56.8% were male. The higher prevalence in males is agreement with data reported in the literature [35,36].

The mean birth weight was 3207.1 gr ± 570.3. These values are within a normal range according to the tables published by the WHO [37]. In the sample, 55.4% were firstborns. Hutchison et al. (2009), on a sample of 287 subjects treated for PP, found that 47.2% were firstborns [38], a lower percentage than in our study. However, Cruchten et al. (2021) found more firstborn babies in a similar sample (60.9%) [36]. A total of 13.5% of the babies were born in a multiple birth. This percentage is quite similar to that found in a previous study (15.3%) [39], but higher than in the general population [40]. A total of 21.6% of the babies were premature, with this percentage being higher than the values of 17.4% [36], 18% [24], and 14.3% [39] found in previous studies.

Instrumental delivery was registered in 20.3%. This percentage is higher than those registered in previous studies: 12% [39] and 13.3% [38].

Leung et al. (2018) associated the total time in supine position during the day with the development of PP [41]. Transporting a baby in a baby backpack instead of in a pushchair can reduce the baby’s supine time. In the present study, only 5.4% of the babies were transported in a baby backpack. In addition, the daily time in prone position at 1 month was only 5.8 min ± 12.3, while the recommendation is for at least 30 to 60 min/day [34]. We did not find data on daily time in prone position in similar samples in the literature, but Vlimmeren et al. (2007) associated tummy time when awake < 3 times per day with an increased risk of PP [42].

In the present study, a total active cervical rotation of 126.3°± 21.9, a right active cervical rotation of 66.3° ± 16.2, and a left active cervical rotation of 60.0° ± 14.9 were registered. We did not find previous data of active cervical rotation in babies with ages similar to ours. Using an inclinometer, Arbogast et al. found a total active cervical rotation of 136.9° in children from 3 to 5 years old (right rotation: 68.1°; left rotation: 68.8°) [43]. In the same study, with videography, the results were 79.5° to the right and 81.9° to the left, with 161.4° in total. The ROM in cervical passive rotation was previously measured in healthy newborns [44]. An average of 189.1° in a sample of 155 healthy newborns was registered [44]. It is generally accepted that a passive rotation of 110° in each direction is normal in healthy babies [31,45]. According to these values, it might be considered that the babies of the present study showed some restrictions in the active cervical rotation ROM, especially toward the left side.

A total of 64.9% of the babies showed a flattening of the right posterior side, and 35.1% showed flattening of the left posterior side of the head. Ballardini et al. (2018) found a predominance of the flattening of the right side in 64.5% of the cases, which is very similar to our finding, in a sample of 107 babies with PP [46].

The mean CVAI was 7.5% ± 3.3. The plagiocephaly severity scale according to the CVAI is: level 1: <3.5%; level 2: 3.5 to 6.25%; level 3: 6.25 to 8.75%; level 4: 8.75 to 11.0%; level 5:> 11.0% [30]. Therefore, the babies in this study presented a moderate deformity, with less severity than that found in previous studies in similar samples (9.14% ± 2.98) [24].

Greater severity of plagiocephaly was related in our study to the transport of the baby in a pushchair, independently of active cervical rotation ROM. To prevent and minimize the progression of PP, it has been recommended that, during the newborn period, when the skull is maximally deformable, the baby should spend minimal time in seats that maintain supine positioning [34]. The results of our study reinforce the evidence for this recommendation. We have not found previous studies that analyzed the relationship between plagiocephaly severity and the transport type of the baby to support our results.

Lower left active cervical rotation ROM was related in our sample with higher severity of PP. Left active cervical rotation ROM was lower than right active cervical rotation and was below the normalized values. Mobility restrictions of the active cervical rotation, at least to one side, have been related with PP [15]. According to Hautopp et al. [47], congenital muscular torticollis is diagnosed when this restriction on one of the sides reaches an asymmetry greater than 15°. Less cervical rotation can favor that, in supine position, babies do not change their headrest on the occiput from one side to the other, and gravitational forces may maintain or aggravate PP [34].

The findings of our study are subject to limitations. They are limited by the cross-sectional design, which was used in a single geographical region. Associations were examined, but no causal relationship can be established. Future prospective studies with previous stratification of the analyzed variables might support our findings due to the low percentage observed in the baby backpack transport category. However, the current findings may be relevant in supporting actual recommendations for babies with PP.

## 5. Conclusions

An increased severity of PP was related with being transported in a baby pushchair; babies should spend minimal time in seats that maintain supine positioning.

Increased severity of PP was also related to a reduced active cervical rotation ROM toward the most restricted side. This fact illustrates the previously established relationship between the limitation of active cervical rotation and the presence of plagiocephaly.

## Figures and Tables

**Table 1:** Descriptive statistics.

Descriptive Statistics		
Babies with PP (*n* = 74)		Descriptive *n* (%)/Mean (SD)
Gender	Females	32 (43.2%)
	Males	42 (56.8%)
Age (weeks)		16.8 (5.0)
Birth weight (gr)		3207.1 (570.3)
Firstborn		41 (55.4%)
Multiple birth		10 (13.5%)
Prematurity		16 (21.6%)
Instrumental delivery		15 (20.3%)
Transport type	Pushchair	70 (94.6%)
	Baby backpack	4 (5.4%)
Time in prone position at 1 month (min)		5.8 (12.3)
Total active cervical rotation ROM (°)		126.3 (21.9)
Right active cervical rotation ROM (°)		66.3 (16.2)
Left active cervical rotation ROM (°)		60.0 (14.9)
Plagiocephaly side	Right	48 (64.9)
	Left	26 (35.1)
CVAI (%)		7.5 (3.3)

The GLM validation indicated no problems. The numerical outputs of the model’s effects are given in Table 2, Table 3 and Table 4. Only the main effect of transport type was significant (*p* < 0.001) in the model including total active cervical rotation ROM as a covariate. According to the estimate, being transported in a pushchair increased the CVAI by 0.557% (Table 2).

**Table 2:** Model effects with the total active cervical rotation ROM as a covariate.

Dependent Variable: CVAI	Estimate	Standard Error	Wald 95% Confidence Interval. Lower Bound/Upper Bound	Wald Chi-Square Statistic	*p* Value
Constant	2.085	0.4977	1.110/3.060	17.551	<0.001
Sex	−0.042	0.1080	0.254/0.170	0.152	0.696
Prematurity	0.062	0.1802	−0.291/0.415	0.119	0.731
Instrumental delivery	0.112	0.1239	−0.131/0.354	0.812	0.368
Firstborn	−0.016	0.1053	−0.223/0.190	0.023	0.878
Multiple birth	0.022	0.1868	−0.344/0.389	0.014	0.905
Transport type: Pushchair	0.557	0.1319	0.298/0.815	17.815	<0.001
Age (weeks)	−0.003	0.0101	−0.022/0.017	0.068	0.794
Birth weight (gr)	−0.00001232	0.0001	0.000/0.000	0.008	0.930
Time in prone position at 1 month (min)	−0.003	0.0034	−0.010/0.004	0.776	0.378
Total active cervical rotation ROM (°)	−0.004	0.0029	−0.010/0.002	2.029	0.154

Only the main effect of transport type was significant (*p* < 0.001) in the model including the right active cervical rotation ROM as a covariate. According to the estimate, being transported in a pushchair increased the CVAI by 0.582% (Table 3).

**Table 3:** Model effects with the right active cervical rotation ROM as a covariate.

Dependent Variable: CVAI	Estimate	Standard Error	Wald 95% Confidence Interval. Lower Bound/Upper Bound	Wald Chi-Square Statistic	*p* Value
Constant	1.659	0.4382	0.800/2.518	14.337	<0.001
Sex	−0.072	0.1079	−0.284/0.139	0.448	0.503
Prematurity	0.146	0.1726	−0192/0.485	0.717	0.397
Instrumental delivery	0.116	0.1223	−0.124/0.356	0.900	0.343
Firstborn	−0.103	0.0990	−0.297/0.091	1.086	0.297
Multiple birth	0.083	0.1925	−0.294/0.460	0.186	0.666
Transport type: Pushchair	0.582	0.1239	0.339/0.825	22.050	<0.001
Age (weeks)	−0.009	0.0097	−0.028/0.010	0.952	0.329
Birth weight (gr)	−0.00002403	0.0001	0.000/0.000	0.033	0.857
Time in prone position at 1 month (min)	−0.003	0.0036	−0.010/0.004	0.611	0.434
Right active cervical rotation ROM (°)	0.001	0.0031	−0.005/0.007	0.114	0.735

The model including the left active cervical rotation ROM as a covariate showed significant effects for transport type (*p* < 0.001) and left active cervical rotation ROM (*p* = 0.034). According to the estimate, being transported in a pushchair increased CVAI by 0.619%, and every increment of one degree in the left active cervical rotation ROM diminished the CVAI by 0.006% (Table 4).

**Table 4:** Model effects with the left active cervical rotation ROM as a covariate.

Dependent Variable: CVAI	Estimate	Standard Error	Wald 95% Confidence Interval. Lower Bound/Upper Bound	Wald Chi-Square Statistic	*p* Value
Constant	2.043	0.4102	1.239/2.847	24.813	<0.001
Sex	−0.083	0.1016	−0.282/0.116	0.669	0.413
Prematurity	0.099	0.1679	−0.230/0.428	0.347	0.556
Instrumental delivery	0.105	0.1188	−0.127/0.338	0.787	0.375
Firstborn	−0.050	0.1030	−0.252/0.152	0.232	0.630
Multiple birth	−0.015	0.1763	−0.360/0.331	0.007	0.933
Transport type: Pushchair	0.619	0.1262	0.372/0.886	24.050	<0.001
Age (weeks)	−0.006	0.0096	−0.025/0.013	0.410	0.522
Birth weight (gr)	−0.00003435	0.0001	0.000/0.000	0.060	0.807
Time in prone position at 1 month (min)	−0.003	0.0031	−0.009/0.003	1.251	0.263
Left active cervical rotation ROM (°)	−0.006	0.0029	−0.012/0.000	4.485	0.034

## Data Availability

The datasets analyzed during the current study are available from the corresponding author on reasonable request. All data analyzed during this study are included in this published article.
